# Advantages of Amplifluor-like SNP markers over KASP in plant genotyping

**DOI:** 10.1186/s12870-017-1197-x

**Published:** 2017-12-28

**Authors:** Satyvaldy Jatayev, Akhylbek Kurishbayev, Lyudmila Zotova, Gulmira Khasanova, Dauren Serikbay, Askar Zhubatkanov, Makpal Botayeva, Aibek Zhumalin, Arysgul Turbekova, Kathleen Soole, Peter Langridge, Yuri Shavrukov

**Affiliations:** 1 Faculty of Agronomy, S. Seifullin Kazakh AgroTechnical University, Astana, Kazakhstan; 2 School of Biological Sciences, Flinders University, Bedford Park, SA Australia; 3 School of Agriculture, Food and Wine, University of Adelaide, Urrbrae, SA Australia

**Keywords:** Amplifluor, Gene-specific primers, KASP, Single nucleotide polymorphism, SNP markers, Universal probes

## Abstract

**Background:**

KASP (KBioscience Competitive Allele Specific PCR) and Amplifluor (Amplification with fluorescence) SNP markers are two prominent technologies based upon a shared identical Allele-specific PCR platform.

**Methods:**

Amplifluor-like SNP and KASP analysis was carried out using published and own design of Universal probes (UPs) and Gene-specific primers (GSPs).

**Results:**

Advantages of the Amplifluor-like system over KASP include the significantly lower costs and much greater flexibility in the adjustment and development of ‘self-designed’ dual fluorescently-labelled UPs and regular GSPs. The presented results include optimisation of ‘tail’ length in UPs and GSPs, protocol adjustment, and the use of various fluorophores in different qPCR instruments. The compatibility of the KASP Master-mix in both original and Amplifluor-like systems has been demonstrated in the presented results, proving their similar principles. Results of SNP scoring with rare alleles in addition to more frequently occurring alleles are shown.

**Conclusions:**

The Amplifluor-like system produces SNP genotyping results with a level of sensitivity and accuracy comparable to KASP but at a significantly cheaper cost and with much greater flexibility for UPs with self-designed GSPs.

**Electronic supplementary material:**

The online version of this article (10.1186/s12870-017-1197-x) contains supplementary material, which is available to authorized users.

## Background

Low- and high-throughput technologies based on SNPs (Single nucleotide polymorphism) are a booming sector in the field of plant genotyping. There are several types of SNP analysis with very different principles and applications (Reviewed in [1–7]). A revolutionary new technology was realised in the commonly named ‘Allele-specific PCR’ (AS-PCR) based on the Fluorescence (or Förster) resonance energy transfer (FRET) method [8]. The main advantage of the AS-PCR method is in the combined application of two components for amplification with: (1) Universal probes (UPs) dually labelled with two fluorophores, and (2) Non-labelled gene-specific primers (GSPs) with a 3′-end designed to match with a SNP, triggering amplification with either one of two fluorescently labelled probes. In earlier reports, the UPs were defined as ‘Universal energy-transfer labelled primers’ [9–11]. However, to avoid any further confusion between ‘probes’ and ‘primers’, we will use only the term ‘Universal probes’ as the synonym of those mentioned above.

### KASP and Amplifluor SNP analyses

Two well-established technologies are based upon a shared AS-PCR platform: (1) KASP or KASPar (Kompetitive Allele Specific PCR or KBiosience Competitive Allele Specific PCR), and (2) Amplifluor (Amplification with fluorescence) SNP markers.

KASP markers are designed and fully operated by LGC Genomics (www.lgcgroup.com), who manufacture and apply the technology through their own proprietary high-throughput robotic system, starting from leaf samples until the finished genotyping results. The basic principles of KASP are similar to those in Amplifluor, but the underlying chemistry and probe/primer assay has been never disclosed, representing a ‘trade secret’ of the Company. A researcher has three options when conducting KASP marker analysis. Large Universities and Research Centres can buy whole or in-part, the instrumental modules for KASP analysis including the reagent assays from LGC Genomics; which obviously represents a substantial investment. The second option is to submit DNA samples for KASP analysis directly to LGC Genomics office, which is significantly cheaper, and costs about 10 times less than other approaches such as the TaqMan method [12]. The final option for the researcher is to order KASP Master-mix and carry out their own genotyping using self-designed GSPs. This method is more popular, since it gives much more flexibility for researchers in the development of various GSPs. The limitation of this option however, is the monopoly on the supply of KASP Master-mix, produced by LGC Genomics, which is relatively expensive, especially for small experiments. Usually, results of KASP markers are highly accurate and effective, whether researchers have bought Robotic lines and KASP Master-mix or simply submitted DNA samples as customers, and received automated KASP genotyping results [13].

The Amplifluor SNP genotyping system was developed by Millipore, the company recently merged with Merck (http://www.merckmillipore.com), and is available as reagent assays or a service. In contrast to KASP, the chemistry, design and sequences of all components in Amplifluor systems were openly disclosed in both an on-line booklet and published papers (for example, [9, 10, 14, 15]). Using this platform, a researcher has the option to either order reagents/service at Millipore or to design and order their own Amplifluor-like system guided by simple instructions, which drops the cost by 10–20-fold compared to KASP markers. Very low cost is one of the most important, but not the sole advantage of ‘self-made’ Amplifluor-like SNP markers. A researcher has much more flexibility to design, test, re-design (if required) and analyse as many SNP primers as they wish using a once-ordered stock of two UPs labelled with separate dyes. In fact, in new experiments, a researcher needs to order only new GSPs at the cost of regular oligos. In this case, the target, accuracy and reproducibility of Amplifluor-like markers are placed completely ‘in the minds’ and ‘in the hands’ of researchers.

### Principles of Amplifluor universal probe structure

**Fig. 1:**
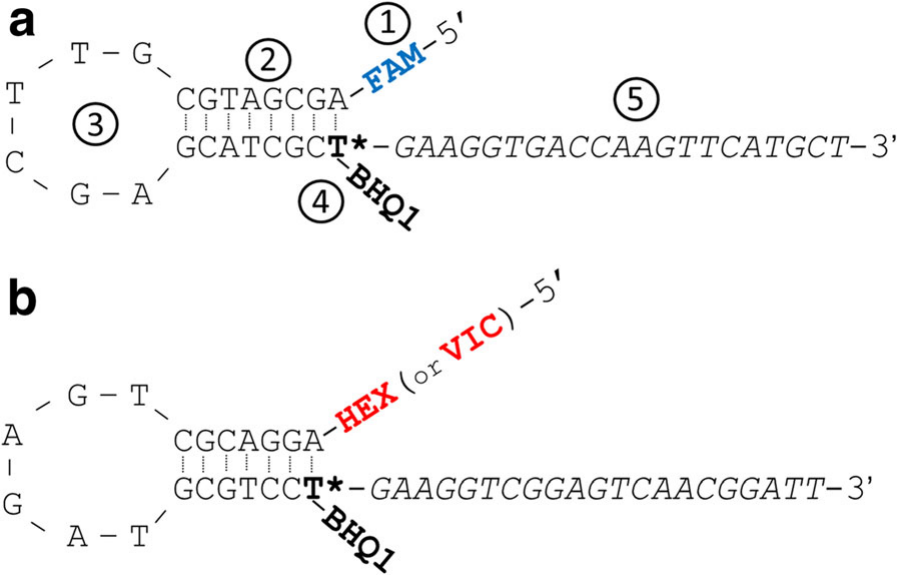
Structure of two Universal probes (UPs) used in Amplifluor SNP markers. Two UPs labelled with FAM (**a**) and with HEX/VIC (**b**) are shown. The five major elements of each UP are indicated by numbers in turn: (1) Fluorophores at the 5′-end; (2, 3) Hairpin with stem and loop, respectively; (4) Modified oligonucleotide Thymine (T) indicated by the asterisk, with Black hole quencher 1 (BHQ1), shown in Bold; (5) A ‘tail’ specific for each UP in the 3′-end, shown in Italics. Figure modified and adapted from [9, 10]

The basic design of UPs was published earlier [9, 10]. Each UP contains: (1) a specific fluorophore on the 5′-end; (2) a stem and (3) loop of a hairpin; (4) a modified nucleotide Thymine (T) with a quencher; and (5) a specific ‘tail’ on the 3′-end corresponding to the fluorophore (Fig. 1). Two fluorophores are used for the labelling of UPs to produce a mixture of two specific UPs in each PCR, where the amplification takes place differentially depending on SNPs and GSPs (Fig. 1a and b).

### Fluorophores

The fluorophore FAM is commonly used in all FRET-based technologies [9]. The choice of the second fluorophore is more variable; historically it was SR [10, 11] but this was later replaced by JOE [3, 14, 15]. HEX remains a popular second fluorophore choice [16], currently used in all KASP markers [17]. The channels in qPCR instruments manufactured by ABI-ThermoFisher were specifically designed for the fluorophore VIC [18]. Details of all fluorophores (FAM, SR, JOE, HEX and VIC), suitable for SNP analyses, are provided in Additional file 1.

### Hairpins

The size and length of the hairpin loop and stem of the UPs have been reported as quite variable for Amplifluor and self-designed Amplifluor-like SNP [9]. However, this was later optimised and the most suitable parameters for different types of SNP analyses were published [10, 15], and are identical to those presented in Fig. 1. Nevertheless, further modifications or even completely new designs for hairpin structures in the UPs are possible by researchers pending the further proven efficiency of such modifications.

### Quenchers

Initially, only DABSYL quencher was used in early Amplifluor SNP marker development [9]. With maximum absorbance at 474 nm, DABCYL is excellent when paired with FAM but it is less suitable for other fluorophores due to its lower absorbance emission spectra (Additional file 1). Nevertheless, successful applications of DABSYL in SNP analysis using FAM and SR [10, 11], FAM and JOE [14, 15, 19, 20], and FAM and HEX [16] have been reported.

Black hole quenchers, BHQ1, BHQ2 and BHQ3, were developed and used in plant pathology with TaqMan PCR [21, 22] and in medical research [23]. Only BHQ1 has the maximum absorbance in the spectra of all fluorophores, FAM, JOE, VIC and HEX (Additional file 1). BHQ1 has been successfully used in our previous studies of Amplifluor-like SNP markers in bread wheat [24].

### 3′-specific tails

On the 3′-end, each of the two UPs has a specific 21 bp-long tail. The first six nucleotides in the tail following the modified T carrying the quencher, are identical. The last 15 nucleotides in the 3′-end are unique for each tail. Initially, shorter tails were used for UPs labelled with FAM and DABSYL quencher [9]. In later papers, tails 1 and 2 were indicated and used for Amplifluor SNP analysis [10, 11, 14, 15] and these two tails are identical to those shown in Fig. 1.

### Design of non-labelled gene-specific primers (GSPs)

A set of three non-labelled GSPs must be designed for each specific Gene of Interest (GOI), following principles that were published earlier [10, 11, 14, 15]. Two forward primers are designed with 3-ends that separately match the two identified variants of the SNP nucleotide. The two forward primers therefore differ by only a single nucleotide at their 3′-end. The single common reverse primer is designed to generate an amplicon with an optimum size between 50 and 120 bp. Each forward primer with SNP-specific 3′-ends must also have a tail-extension on their 5′-end. These extensions are identical (but not complementary) to tails 1 and 2 in the two corresponding UPs, presented in Table 1. The set of three GSPs required are regular non-labelled oligonucleotides, costing the same as ordinary primers purchased for routine PCR, used in all molecular biology laboratories.

**Table 1:** Sequence of tails and Universal probes (UPs) and an example of Gene specific primers (GSPs), used in Amplifluor-like SNP markers

Name	Sequence (5′-3′)
A. Tails	
Tail 1-(FAM)	GAAGGTGACCAAG*TTCATGCT*
Tail 2-(HEX/VIC)	GAAGGTCGGAGTC*AACGGATT*
Tail 3-(FAM)	G*TTCATGCT*
Tail 4-(HEX/VIC)	G*AACGGATT*
B. Universal probes (UPs)	
UP-1 (FAM)	FAM-AGCGATGCGTTCGAGCATCGC(T*) *GAAGGTGACCAAGTTCATGCT*
UP-2 (HEX/VIC)	HEX/VIC-AGGACGCTGAGATGCGTCC(T*) *GAAGGTCGGAGTCAACGGATT*
UP-3 (FAM)	FAM-AGCGATGCGTTCGAGCATCGC(T*) *GTTCATGCT*
UP-4 (HEX/VIC)	HEX/VIC-AGGACGCTGAGATGCGTCC(T*) *GAACGGATT*
C. Gene-specific primers (Example of barley KATU37, Contig ABC08184)	
KATU37-F1	*GAAGGTGACCAAGTTCATGCT*ATTGAGCGATTACGACGAGC
KATU37-F2	*GAAGGTCGGAGTCAACGGATT*ATTGAGCGATTACGACGAGA
KATU37-R	GCAAGGACAAGACAGGCAG
KATU37-F3	*GTTCATGCT*ATTGAGCGATTACGACGAGC
KATU37-F4	*GAACGGATT*ATTGAGCGATTACGACGAGA
KATU37-R	GCAAGGACAAGACAGGCAG

In two pairs of tails, the identical sequences are underlined either with normal case or in Italics. In UPs and in GSPs, the tails are shown in Italics. In UPs, fluorophores are located in the 5′-end and the Thymidine (T) asterisked indicates the positions modified with quencher BHQ-1. Data were extracted from previously published material [10, 11, 14, 15] with modifications and additional design

### PCR protocols for Amplifluor SNP and KASP markers

There are many different published protocols suitable for Amplifluor SNP and KASP markers, using various PCR thermal cyclers. Protocols for Amplifluor SNP markers contain either 35–40 identical cycles [10, 11, 19, 20] or two groups of 20–25 cycles [14, 15]. Protocols for use with KASP Master-mix contain 10 cycles with decreased gradient temperature with a further 25–35 cycles [25–31]. Details of all protocols are present in Additional file 2.

### Compatibility of KASP with ‘home-made’ master-mixes

As was mentioned above, researchers working with KASP markers can buy and use the entire robotic line for SNP genotyping various crops, as for example in the University of Adelaide, Australia. Others submit DNA samples to the LGC office and publish the received SNP genotyping results, as shown for barley [32] and peanut [33].

However, many researchers have also ordered KASP Master-mix and carried out SNP genotyping in their own laboratory using regular thermal cyclers or qPCR instruments, as for example in wheat [26, 28, 34, 35], soybean [27, 29, 36] and maize [37]. Interestingly, in these papers, it was mentioned that GSPs, used together with KASP Master-mix, had exactly the same tails 1 and 2, as shown in Table 1. Therefore, the two different types of GSPs, developed for KASP and Amplifluor-like analyses, have identical tails. There is no available information about the complete UPs structure in KASP, but two tails appear to be identical to those used in Amplifluor SNP markers as shown in Fig. 1. We can speculate, therefore, that commercially produced KASP Master-mix and self-designed Amplifluor-like SNP markers are compatible and can be used interchangeably.

The aims of this paper are: (1) to estimate the effects of modifications and optimise the protocol for reagents, instruments, Master-mix, PCR, fluorescent signal detection and SNP calls; (2) to evaluate the basic protocol of ‘self-made’ Amplifluor-like markers for SNP analysis in plants compared to those freely available in published articles; (3) to compare the results of genotyping using self-made Amplifluor-like markers and KASP markers targeting the same SNP.

## Methods

### Plant material and DNA extraction

Plants of bread wheat and barley from a wide range of cultivars were grown in a field trial in the Akmola region, Kazakhstan. Leaves were collected and DNA extracted as described in our earlier paper [24] following a Phenol-chloroform extraction method [38], and DNA quality checked by PCR.

### Design of Universal probes (UPs) and gene specific primers (GSPs)

In our experiments, we used UPs with four types of specific tails (Table 1A). The first two tails (1 and 2) were identical to those published earlier [10, 11, 14, 15]. The last two tails (3 and 4) were shorter, designed with nucleotides 2–14 missing from the corresponding tails 1 and 2 (Table 1A). Sequences of the four UPs are presented in Table 1B, where first two Universal probes, UP-1 and UP-2, are identical to those presented in Fig. 1. The last two Universal probes, UP-3 and UP-4, have shortened tails as described. Two sets of GSPs (F1, F2, R and F3, F4, R) were designed and used for each of GOI with the corresponding UP-1/UP-2 and UP3/UP-4, respectively. An example of one such GSP for the barley GOI, Contig ABC08184, is presented in Table 1C and in Additional file 3. All UPs labelled with FAM, VIC or HEX were synthesized by DNA Synthesis Company, Moscow, Russia (http://www.oligos.ru), and all gene-specific primers were synthesized by Biosan Company, Novosibirsk, Russia (http://www.biosan-nsk.ru).

### Amplifluor-like SNP and KASP analysis

A Quant Studio-7 Real-Time PCR instrument, qPCR (ThermoFisher Scientific, Paisley, UK) and FLUOstar Omega Microplate Reader (BMG LabTech, Ortenberg, Germany) were used at the Kazakh Agro-Technical University, Astana, Kazakhstan. A Real-Time qPCR system, Model CFX96 (BioRad, Gladesville, NSW, Australia) was used at Flinders University, Bedford Park, SA, Australia.

The PCR conditions were the same as described in our earlier paper [24] with altered PCR cocktail composition. Microplates with 96- or 384-wells were used with a 10 μl or 5 μl total reaction volume in each well, respectively. The PCR cocktail contained 2 x Master-mix with the following reagents in final concentrations: 1 x PCR Buffer, 1.8 mM MgCl_2_, 0.2 mM each of dNTPs, 0.25 μM each fluorescent label probe, 0.15 μM of each forward primer, 0.78 μM of reverse primer and 0.5 units of *Taq* DNA polymerase (GenLab, Astana, Kazakhstan) or 0.1 units of Maxima Hot-Start *Taq*-polymerase (ThermoFisher, USA). Half of the PCR volume was genomic DNA, adjusted for 10 ng/μl. The PCR program was optimised as presented in Additional file 2. Genotyping with SNP calling was determined automatically, as described earlier [24]. Each experiment was repeated twice and technical replicates confirmed a confidence of SNP calls.

KASP analysis was carried out according to the manufacturers’ protocol in the LGC Genomics high-throughput genotyping line based at the Plant Genomics Centre, the University of Adelaide, SA, Australia. KASP Master-mix and consumable materials were supplied by LGC Genomics (Middlesex, UK).

## Results

### Optimisation of Amplifluor universal probes with different tails

**Fig. 2:**
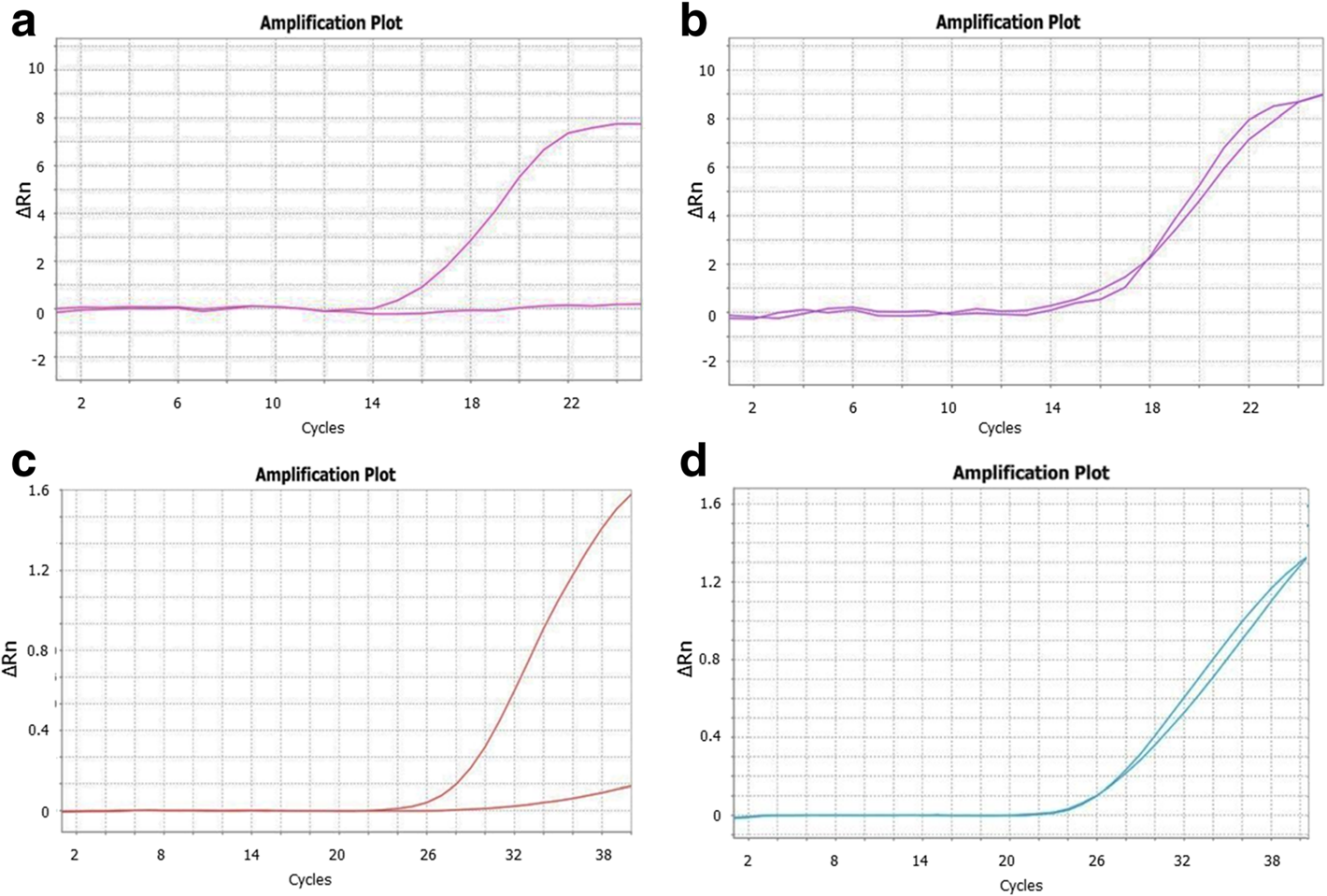
Amplification curves for FAM- and VIC-labelled UPs with different tails. Amplifluor-like SNP markers were developed and used with ‘standard’ tails 1 and 2 for homozygous (**a**) and heterozygous (**b**) genotypes. Two UPs with shorter tails (tails 3 and 4) were used for SNP analysis of the same homozygous (**c**) and heterozygous (**d**) genotypes. The data were extracted from [24] for plants of different bread wheat cultivars from Kazakhstan, genotyped with the Amplifluor-like marker KATU48 designed for a SNP in the *TaDREB5* Transcription factor. Relative amplification units, ΔRn, are show in Y-axis for FAM or VIC fluorescence signals

Amplifications using UPs with 21-bp length tails 1 and 2, and with the same UPs but with 9-bp length tails 3 and 4, revealed similar results using qPCR instruments (Fig. 2). The amplification of fluorescently labelled products using UPs with tails 1 and 2 started much earlier, at cycle 14, in both homo- and heterozygous alleles (Fig. 2a and b). Relative amplification, ΔR_n_, reached 8 units for a single allele amplification, with very strong differentiation between alleles (Fig. 2a). Both heterozygote alleles showed rapid and equal amplification curves with 9 units of ΔR_n_ at the PCR end-point.

In the case of UPs with tails 3 and 4, the amplification started later, at cycle 24, in both homo- and heterozygous alleles (Fig. 2c and d). The discriminations between homozygote alleles were based on a smaller difference in ΔR_n_, reaching only 3 units of relative amplification but still very clear after 30–35 PCR cycles (Fig. 2c).

### Compatibility of different qPCR instruments and fluorophores for allele discrimination

**Fig. 3:**
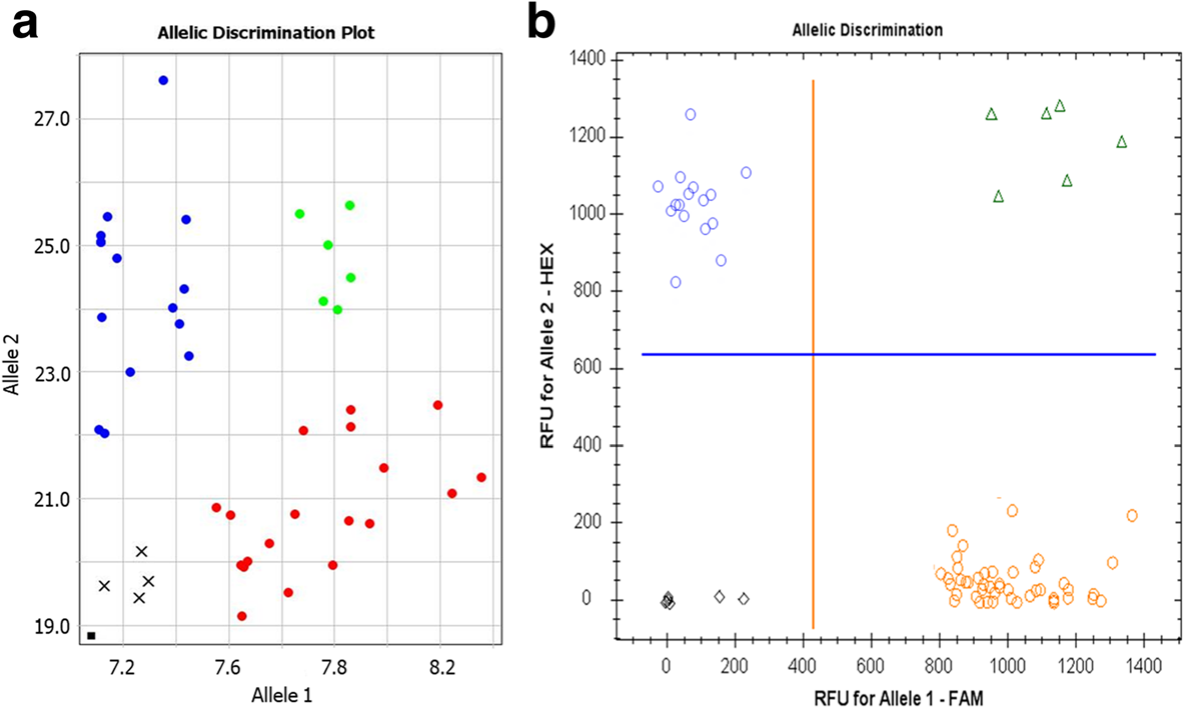
Comparison of Amplifluor-like SNP marker application with the same UPs labelled with FAM and VIC fluorophores. Quant Studio-7 Real-Time PCR instrument (ThermoFisher Scientific, Paisley, UK), designed for FAM and VIC scoring (**a**) and Real-Time qPCR system, Model CFX96 (BioRad, Gladesville, NSW, Australia), designed for FAM and HEX discrimination (**b**), were used. Automatic calls for alleles are shown in different colours, using supplementary software for each instrument separately. X- and Y-axes show Relative amplification units, ΔRn, for FAM and VIC fluorescence signals (**a**); and Relative fluorescence units, RFU, for FAM and HEX (**b**); as determined by the qPCR ThermoFisher and BioRad instruments, respectively

No differences were found in the identical experiments of Amplifluor-like SNP genotyping with UPs labeled with FAM and VIC, using qPCR instruments manufactured by ThermoFisher and BioRad (Fig. 3). These instruments have channels designed for FAM and VIC (Fig. 3a) and FAM and HEX (Fig. 3b) absorption, respectively. Similar results were recorded in our experiments with FAM and HEX-labelled UPs in the same qPCR systems (Data not shown).

### PCR protocols for Amplifluor SNP markers

Three types of protocols were tested in our experiments using various PCR cyclers (Additional file 2). The first two protocols were designed and used for the SNP Amplifluor method. Protocol 1 represents a very generic three-step cycling PCR with a relatively long extension time (40 s) and 35–45 cycles. In protocol 2, two portions of PCR cycles were used with higher/lower annealing temperature and shorter/longer time for annealing/extension in the first 20 and last 22 cycles, respectively. Protocol 3 was published and used for KASP markers with similar biochemistry and was prescribed by LGC Genomics, as the manufacturer of KASP technology.

Our experiments with self-designed Amplifluor-like SNP markers and analyses of various GOI in bread wheat, barley and chickpea revealed no significant differences in the use of any of these three types of PCR protocols (Data not shown). Our self-designed protocol 4 (Additional file 2) is most similar to protocol 2 [14, 15] with the difference being the inclusion of ‘doubled cycles’ combining a higher and lower annealing temperature with shorter and longer annealing/extension time in the first and second part of each doubled cycle, respectively. Figure 2 shows PCR amplification using different Amplifluor-like SNP probes with 20 and 40 cycles, but we found that 30–35 doubled cycles in the latter case was sufficient for clear recognition of allele genotyping. We can conclude in this section that the application of UPs in Amplifluor-like SNP markers is stable regardless of PCR protocol variabilities, therefore researchers may choose from published PCR protocols or design their own to suit their specific needs.

### Adjustment of PCR protocol with Amplifluor-like SNP markers to resolve false-positives for heterozygotes

**Fig. 4:**
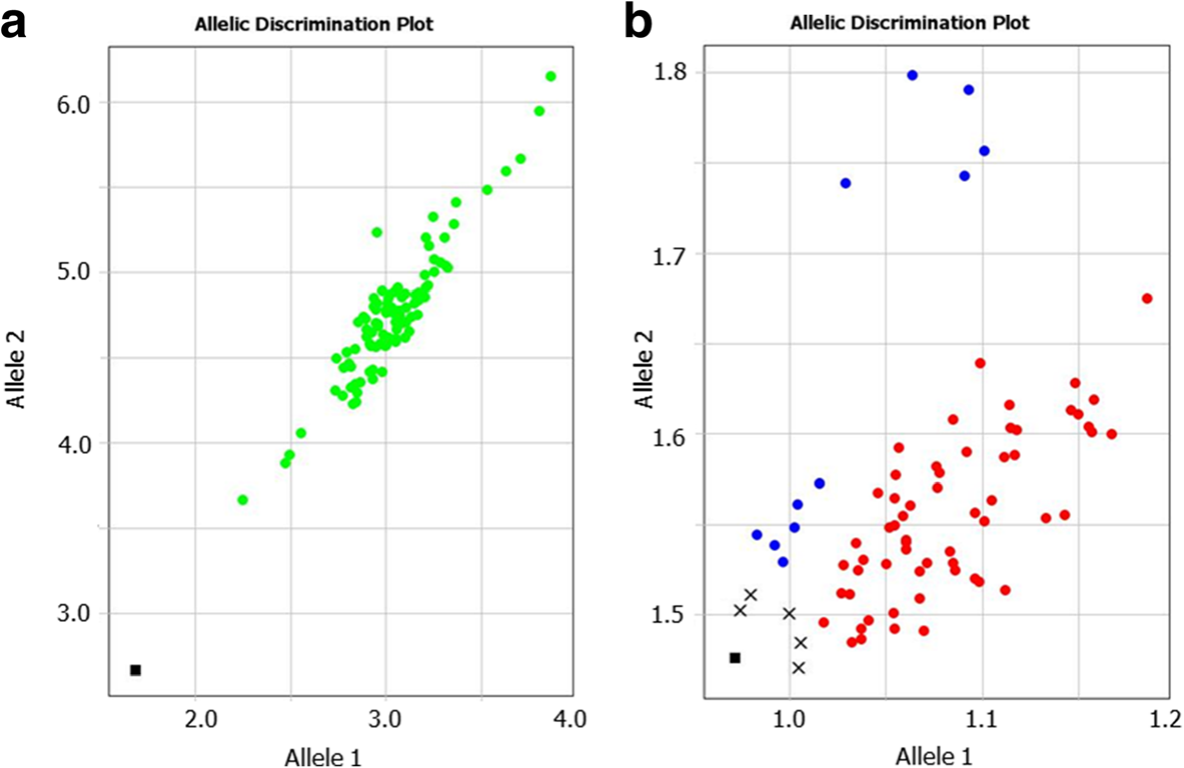
Comparison of different concentrations of *Taq*-polymerase in PCR with Amplifluor-like SNP markers for ‘trouble-shooting’ of allele discrimination. The identicial PCR protocol was applied with the same DNA samples from a wheat collection from Kazakhstan and the same marker W58, using 0.5 units (**a**) or 0.1 units (**b**) of Maxima Hot-start *Taq*-polymerase (ThermoFisher, USA). X- and Y-axes show Relative amplification units, ΔRn, for FAM and VIC fluorescence signals, respectively, as determined by the qPCR instrument

The efficiency and level of amplification play the most important roles in SNP genotyping. Despite the precise allele specificity, Amplifluor-like SNP determination can fail and show ‘false-positive’ results, typically as heterozygotes ‘*ab*’, where those genotypes are absent or present in very small numbers. An example of this false identification of genotypes as heterozygotes is presented in Fig. 4a for a bread wheat germplasm collection from Kazakhstan with the Amplifluor-like SNP marker W58 designed in TF *TaNFYC-A7*. The perfect diagonal line in Fig. 4a shows equal amplification signal with both FAM and VIC fluorescent labels in each studied genotype. This situation also occurred using other developed Amplifluor-like SNP markers. However, this cannot be a true reflection of the situation, since a germplasm collection will mostly contain little to no heterozygotes in any given GOI.

Conflicts resulting in false genotype identifications were resolved simply through reducing the concentration *Taq*-polymerase used. In the case presented in Fig. 4, Maxima Hot-Start *Taq*-polymerase, manufactured by ThermoFisher, was used. The amplification capacity of this *Taq*-polymerase using the typical 0.5 enzyme units per 10 μl of total PCR was so strong that it finally amplified fragments with both fluorescent labels. Figure 4b shows our results using the same bread wheat collection genotypes and the same SNP primer W58, only with five-fold less Maxima Hot-Start *Taq*-polymerase (0.1 enzyme units per 10 μl total PCR reaction). Clear allele discrimination was then observed in the studied wheat accessions, with heterozygotes completely absent and only a few undetermined genotypes (Fig. 4b).

### Comparison of KASP and Amplifliour-like systems with the same Master-mix

**Fig. 5:**
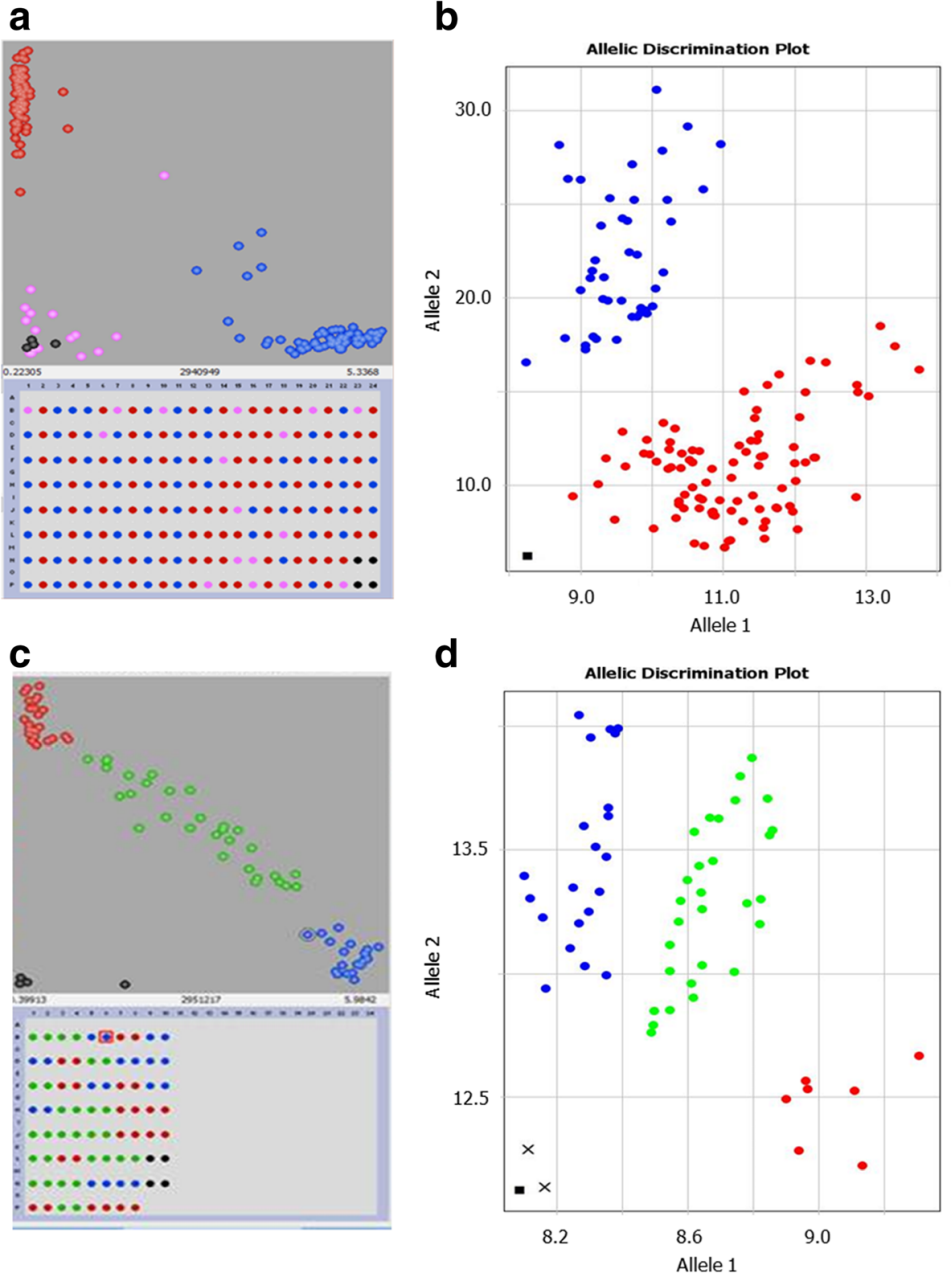
Comparison of allele discrimination in KASP and Amplifluor-like systems using the same Master-mix. SNP genotyping was developed targeting SNPs in a barley collection, in the *FT5* gene using KASP (**a**) and Amplifluor-like (**b**) systems; and in the *PHY-C* gene using the same KASP (**c**) and Amplifluor-like (**d**) systems. Blue and red dots indicate genotypes with homozygous alleles, green dots for heterozygous, pink dots and crosses for samples with no signals, and black dots and squares shows NTC, No template controls. X- and Y-axes show Relative amplification units, ΔRn, for FAM and HEX/VIC fluorescence signals, respectively, as determined by the qPCR instrument

The same KASP Master-mix was used in both the original KASP and in the self-developed Amplifluor-like systems. Figure 5 shows examples of two GOI studied in barley, where the same KASP Master-mix was used with different GSPs and instruments for amplification and allele discrimination. Paired panels a/b and c/d in Fig. 5 show the very similar output from both systems, the only difference being the design of the screens and colour variation for allele discrimination, related to the technical settings of the instruments.

### Application of Amplifluor-like SNP for rare allele genotyping

**Fig. 6:**
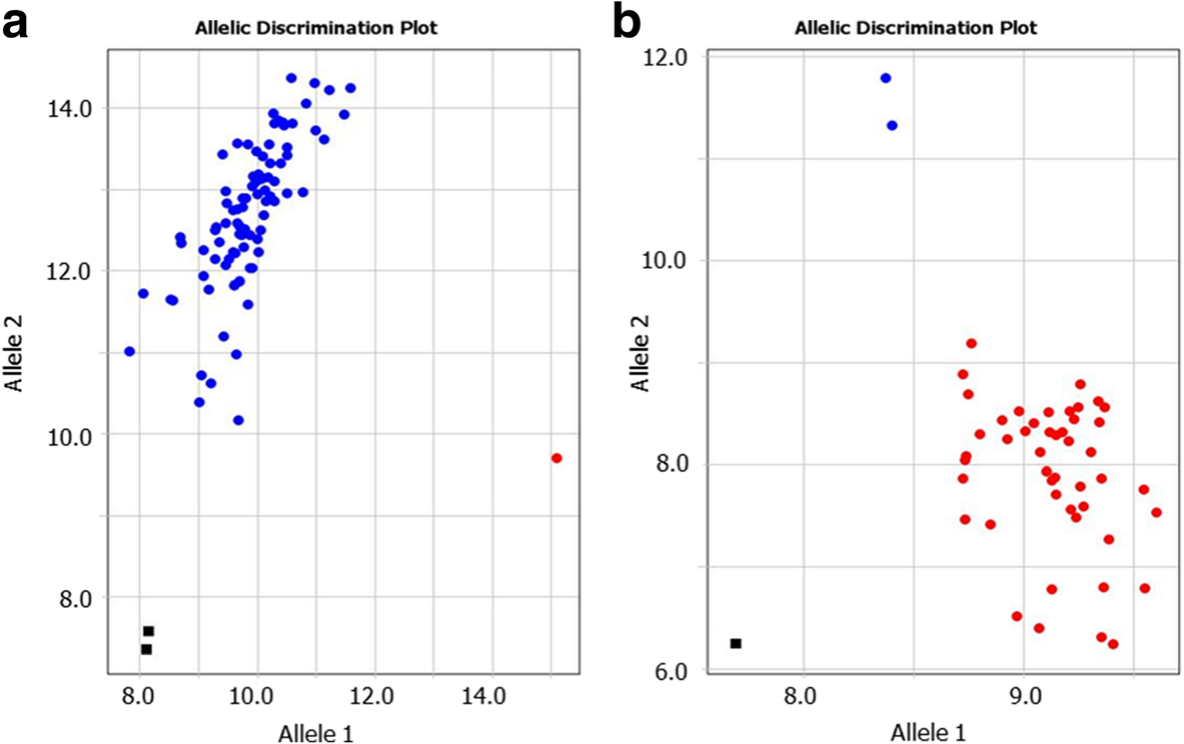
Examples of rare SNP alleles in bread wheat and barley originated from Kazakhstan. **a** Rare SNP allele 1 in the collection of Kazakh wheat (labelled with FAM). **b** Results of the discrimination of rare SNP allele 2 (labelled with VIC) in the Kazakh barley collection. X- and Y-axes show Relative amplification units, ΔRn, for FAM and VIC fluorescence signals, respectively, as determined by the qPCR instrument

Examples of genotyping scores in rare alleles both in Kazakh wheat and barley are presented in Fig. 6. These results were generated using an Amplifluor-like system with our own self-designed GSPs.

## Discussion

KASP and Amplifluor-like markers have similar principles and applications for SNP genotyping. This point is supported by the available information showing that tails 1 and 2 in GSPs are identical in both methods. Additionally, previously published information in conjunction with the data presented in this paper confirms that KASP Master-mix can be used with both original KASP technology and a self-developed Amplifluor-like system for SNP genotyping. The structure of UPs in the KASP system remains undisclosed, but there is mention of ‘two universal FRET cassettes’ with fluorophores (FAM and HEX) on the web-site of LGC Genomics [17]. There is also no available information about the type of quencher in the UPs, which remains unknown for KASP genotyping, but we can speculate that the general structure of UPs in both KASP and Amplifluor-like systems are very similar, if not identical.

The perfect optimisation of UPs and associated well-organised business and marketing makes KASP markers very popular among plant biologists and the wider range of researchers. A large and ever-growing number of publications with references to KASP analyses indicates a continued high interest in this technology (Table 2). The cost of KASP markers, however, remains relatively high, even when ordering and using KASP Master-mix in researcher’s laboratory. In contrast, Amplifluor-like self-developed SNP markers share the same molecular platform but are much cheaper compared to KASP. The fewer number of publications indicates that scientists may be unaware of the Amplifluor-like SNP system and, therefore, submit samples for KASP analyses or order KASP Master-mix rather than develop and use their own Amplifluor-like markers (Table 2).

**Table 2:** Comparison of KASP and Amplifluor marker characteristics, cost and publications with references to these methods

	KASP	Amplifluor
Automatic/robotic system	Low- and high-throughput	Possible but not included
Sequence of probes and primer design	Unavailable	Available
Cost of low- and high-throughput equipment line (US$)	450 K–550 K	N/A
Cost of reagents in bulk per reaction, 5 or 10 μl (US$)	3.0–6.0	0.06–0.12
Number of publications in Scopus database (including those with plants)	151 (61)	27 (5)

Our results indicate that it is not complicated to order UPs based on published sequences or own design. The two UPs, shown in Fig. 1 and Table 1, contain a quencher in the middle of the probe and different dyes on the 5′-ends. Therefore, the UPs are relatively expensive reagents. However, these two UPs are common for all experiments, and so need only be ordered once in bulk. They can then be used in all further AS-PCR experiments for a considerable time without limitations, which significantly drops the cost. The similar KASP Master-mix with UPs is more expensive. GSPs, with examples of sequences in Table 1 and Additional file 3, can be simply ordered as regular oligonucleotides.

SNPs can be identified through the researcher’s own experiments, or sourced from published papers or publicly available databases. Depending on the targets of a researcher, a SNP may be required within a GOI or in the flanking regions. It is strongly recommended that the genetic fragment with putative SNPs is sequenced to verify it’s presence, and the sequenced genotypes showing SNPs can then be used as References for Amplifluor-like or KASP methods for SNP analysis. However, this is not always possible, due to timing or financial limitations, particularly in small laboratories. In this case, it is possible to make a preliminary analysis of the putative SNPs based on published results or SNP databases. The advantage of UPs provides extra-opportunity to order and screen as many putative SNPs as researchers wish, without sequencing requirements and with fewer expenses. However, technical calls for allele discrimination from the instruments used must be verified by later sequencing of genotypes showing the clearest SNP scoring results. After SNP verification, such Reference genotypes can be used for further genotyping.

In Fig. 3 we showed the application of the same self-developed Amplifluor-like SNP markers in two different instruments. The Real-time qPCR systems were manufactured by ThermoFisher and BioRad, respectively. However, any similar qPCR cycler can be used for the analysis of Amplifluor-like SNP markers, if such instruments have at least two channels for absorbance analyses in two different spectra compatible with the chosen fluorophores [39, 40].

At the same time, most current researchers, particularly when using KASP Master-mix, do not need to use any Real-time qPCR systems at all. Regular PCR can be used, with any ordinary PCR cycler, including older models. PCR products can be analysed using a spectrophotometer with absorption of the wavelength corresponding to the fluorophores used (Additional file 1), which can then be converted manually into SNP allele discrimination.

However, it is much more productive and appropriate to use a Microplate Reader when high-throughput methods are required. In this case, 96- and 384-well plates are the most suitable format, sealed by optically clear tapes and directly screened by a Microplate Reader after completion of PCR. A large range of manufactured Microplate Readers gives the opportunity for researchers to choose the most appropriate product depending on manufacturer price, availability and personal preferences. In published papers, SNP Amplifluor and KASP markers are mostly scored using the following Microplate Readers: Victor 1420, Perkin-Elmer Wallac, USA [11], FLUOstar Optima or Omega, BMG LabTec, Germany [20, 25], and Tecan Safire, Tecan, Switzerland [28, 36].

Three types of protocols were tested in our experiments, using various PCR cyclers (Additional file 2). The first two protocols were designed for and used with the SNP Amplifluor method. Protocol 1 represents a very generic three-step cycling PCR with a relatively long extension time (40 s) and 35–45 cycles. In Protocol 2, two portions of PCR cycles were used with higher/lower annealing temperature and shorter/longer time for annealing/extension in the first 20 and last 22 cycles, respectively. This produces more specific amplification of the PCR product in the beginning, but more enhanced amplification and extension in the second part of the protocol. Protocol 3 was published and used for KASP markers with similar biochemistry and was prescribed by LGC Genomics, the manufacturer of KASP technology. This protocol contains an initial 10 cycles, of a relatively long duration (60 s) with a gradient of temperatures. Such a combination of long annealing and declining temperature in each cycle in Protocol 3 allows amplification with maximal variability in the initial PCR steps. The following cycles have 60 s annealing with no dedicated extension steps. The note made in [26, 28] that “amplicons are smaller than 120 bp, and extension step is unnecessary” is correct at this point, but it conflicts with the necessity of a very long previous annealing step (60 s). In general PCR settings, 10 s is sufficient for annealing of such amplicons. However, the mentioned note matches perfectly the total time for annealing + extension in Protocols 1 and 2, which was exactly the same (60 s) as in Protocol 3. We believe that such long time for annealing + extension is required due to the similar nature of SNP Amplifluor-like and KASP markers, including UPs and GSPs. In our experiments, the most optimal PCR conditions were those described in Protocol 4 (Additional file 2) with 20 ‘doubled cycles’ for UPs with tails 1 and 2, and with 30–40 cycles with tails 3 and 4 (Fig. 2).

The PCR protocol must be carefully adjusted, with a particular focus on the efficiency and level of amplification using whichever brand of *Taq*-polymerase is preferred. The main aim is to achieve a gentle start in the amplification curve from a single fluorescent label only, and only very minimal amplification from the second fluorescent label. Use of high concentrations of *Taq*-polymerase with strong amplification capacity produces a greater risk of masking true results through false amplification, since both GSPs differ by only a single nucleotide on the 3′-end. Therefore, the adjustment of PCR protocols to incorporate a lower concentration of *Taq*-polymerase and fewer PCR cycles is required to select the optimal start in fluorescence signal amplification. A Reference genotype with a known sequence can be very helpful in the initial PCR protocol adjustments.

SNPs can have differential distributions among studied genotypes. The clearest genotyping results of SNPs occur when large proportions of both alleles occur in the studied accessions. However, this is not always the case and is mainly related to the nature of gene-pool in the studied accessions. For example, advanced breeding lines sharing the same pedigree or a segregating population originating from parents adapted to a local environment, with no introgression of exotic germplasms, very often have limited genetic polymorphism, particularly in the some GOI. Therefore, cases showing a large proportion of both SNP alleles occur infrequently. In contrast, rare SNP alleles, where only one or two genotypes differ from the rest of the studied germplasm collection, or from other offspring in the segregating population, occurred more often. In our studies, up-to 60% of wheat and 30% of barley germplasms originated from Kazakhstan can be characterised by rare SNP alleles. High probabilities of rare SNP alleles can make it especially important for further consideration and application in the crossing program to produce the perfect mapping population with predicted segregating of the GOI. The application of Amplifluor-like system for rare allele identification showed perfect results in our experiments (Fig. 6).

## Conclusions

Our study has shown that the Amplifluor-like system for SNP genotyping delivers results with a sensitivity and accuracy equal to those of KASP at a significantly cheaper cost, and with much greater flexibility in UPs with self-designed GSPs. For screening purposes, when a researcher needs to test and check hundreds or thousands of putative SNPs, the ‘self-made’ Amplifluor-like SNP system is much more suitable for developing simple, cheap and accurate SNP markers. This statement is particularly important for plant genetics and crop breeding, where hundreds and thousands of individuals from progenies or breeding lines must be analysed simultaneously, or during a short time for Marker-assisted selection of the best-performing plants. Furthermore, the ‘self-made’ Amplifluor-like system for SNP genotyping is also most suitable for medical, veterinary and microbiological research. The presented results of this study and application of the Amplifluor-like SNP system can open new avenues for scientists to develop and adapt existing systems of SNP markers for their own additional improvements.

## Additional files

Additional file 1: Names and characteristics of fluorophores and quenchers used in SNP analyses based on FRET principles (PDF 232 kb)

Additional file 2: Protocols used for Amplifluor and KASP markers and basic PCR cyclers. Protocols 1 and 2 were designed for Amplifluor SNP analysis, while Protocol 3 was used for KASP markers. Protocol 4 was designed as optimal and used in the current study. (PDF 152 kb)

Additional file 3: Example of design for non-labelled Gene-specific primers (GSP), KATU37, in barley Contig ABC08184. SNP position in the sequence was coded ‘M’, designating mixed nucleotides ‘A’ and ‘C’, and highlighted in red. Two forward primers and one common reverse primer are shown in Bold and highlighted in blue and purple, respectively. Amplicon size is indicated. Two sets of forward primers with ‘standard’ and short tails (Table 1A), identical to those in the corresponding UPs (Table 1B), and common reverse primer were developed. The tails are shown in normal case. (PDF 296 kb)

## Abbreviations

Amplifluor: Amplification with fluorescence
GSP: Gene-specific primers
KASP: Kompetitive Allele Specific PCR or KBiosience Competitive Allele Specific PCR
SNP: Single nucleotide polymorphism
UP: Universal probes
