# Supplementary material for: Advantages of Amplifluor-like SNP markers over KASP in plant genotyping
Source: BMC Plant Biol. 2017 Dec 28;17(Suppl 2):254. doi: 10.1186/s12870-017-1197-x (PMC5751575; doi:10.1186/s12870-017-1197-x)
Supplement: Supplementary file 1 — Names and characteristics of fluorophores and quenchers used in SNP analyses based on FRET principles (PDF 232 kb) [file 12870_2017_1197_MOESM1_ESM.pdf]

Additional file 1

Names and characteristics of fluorophores and quenchers used in SNP analyses based on FRET principles

**Abbreviated name**	**Full name**	**Absorption, λ_{max} / nm**	**Emission, λ_{max} / nm**	**References**
**Fluorophores**				
FAM	6-Fluorescein Phosphoramidite	494	518	[9]
SR	Sulforhodamine	566	584	[10]
JOE	6-carboxy-4,5-dichloro-2,7-dimethoxyfluorescein	520	548	[15]
HEX	6-carboxy-2',4,4',5',7,7'-hexachlorofluorescein succinimidyl ester	535	556	[16]
VIC	No full name	538	554	[18]
**Quenchers**				
DABSIL	4-(4-dimethylaminophenyl) diazenylbenzoic acid	474	-	[9]
BHQ-1	Black Hole Quencher 1	534	-	[21]
