# Supplementary material for: Advantages of Amplifluor-like SNP markers over KASP in plant genotyping
Source: BMC Plant Biol. 2017 Dec 28;17(Suppl 2):254. doi: 10.1186/s12870-017-1197-x (PMC5751575; doi:10.1186/s12870-017-1197-x)
Supplement: Supplementary file 2 — Protocols used for Amplifluor and KASP markers and basic PCR cyclers. Protocols 1 and 2 were designed for Amplifluor SNP analysis, while Protocol 3 was used for KASP markers. Protocol 4 was designed as optimal and used in the current study. (PDF 152 kb) [file 12870_2017_1197_MOESM2_ESM.pdf]

Additional file 2

Protocols used for Amplifluor and KASP markers and basic PCR cyclers. Protocols 1 and 2 were designed for Amplifluor SNP analysis, while Protocol 3 was used for KASP markers. Protocol 4 was designed as optimal and used in the current study.

Steps	**1**<br>[10-11,19-20]			**2**<br>[14-15]			**3**<br>[25-31]			**4**<br>Current study		
	Temp. (°C)	Time	Cycle	Temp. (°C)	Time (sec)	Cycle	Temp. (°C)	Time	Cycle	Temp. (°C)	Time	Cycle
Initial denaturation	95	3-4 min	1	96	4-10 min	1	94-95	10-15 min	1	95	5 min	1
Denaturation	95	10 sec		95	10-15 sec		94-95	20 sec		95	10 sec	
Annealing	55-60	20 sec		55-64	5-10 sec		61-65, drop 0.6-1°C per cycle	25-60 sec		56-60	10 sec	
Extension	72	40 sec	35-45	72	10-15 sec	20			10	72	20 sec	
Denaturation	-	-	-	96	10 sec		94	10-20 sec		95	10 sec	
Annealing				50-56	20-30 sec		55-60	60 sec		51-55	20 sec	
Extension				72	40 sec	20-25	-	-	25-35	72	40 sec	25-30
