# Supplementary material for: Advantages of Amplifluor-like SNP markers over KASP in plant genotyping
Source: BMC Plant Biol. 2017 Dec 28;17(Suppl 2):254. doi: 10.1186/s12870-017-1197-x (PMC5751575; doi:10.1186/s12870-017-1197-x)
Supplement: Supplementary file 3 — Example of design for non-labelled Gene-specific primers (GSP), KATU37, in barley Contig ABC08184. SNP position in the sequence was coded ‘M’, designating mixed nucleotides ‘A’ and ‘C’, and highlighted in red. Two forward primers and one common reverse primer are shown in Bold and highlighted in blue and purple, respectively. Amplicon size is indicated. Two sets of forward primers with ‘standard’ and short tails (Table 1A), identical to those in the corresponding UPs (Table 1B), and common reverse primer were developed. The tails are shown in normal case. (PDF 296 kb) [file 12870_2017_1197_MOESM3_ESM.pdf]

Additional file 3

Example of design for non-labelled Gene-specific primers (GSP), KATU37, in barley Contig ABC08184. SNP position in the sequence was coded 'M', designating mixed nucleotides 'A' and 'C, and highlighted in red. Two forward primers and one common reverse primer are shown in Bold and highlighted in blue and purple, respectively. Amplicon size is indicated. Two sets of forward primers with 'standard' and short tails (Table 1A), identical to those in the corresponding UPs (Table 1B), and common reverse primer were developed. The tails are shown in normal case.

Sequence:

5'-CATCTTTCGCACGTCTGTTTACAAG**ATTGAGCGATTACGACGAG**M AAGAATG
CGACAGAGATCGTCCTACCTGCACGAGGAGG**CTGCCTGTCTTGTCCTTGC**CGG-3'

PCR product size: 77 bp.

Primers:

KATU37-F1: 5'-GAAGGTGACCAAGTTCATGCT**ATTGAGCGATTACGACGAGC**-3'
KATU37-F2: 5'-GAAGGTCGGAGTCAACGGATT**ATTGAGCGATTACGACGAGA**-3'
KATU37-R: 5'-**GCAAGGACAAGACAGGCAG**-3'

KATU37-F3: 5'-GTTCATGCT**ATTGAGCGATTACGACGAGC**-3'
KATU37-F4: 5'-GAACGGATT**ATTGAGCGATTACGACGAGA**-3'
KATU37-R: 5'-**GCAAGGACAAGACAGGCAG**-3'
